# Can Serum-Specific IgE/Total IgE Ratio Predict Clinical Response to Allergen-Specific Immunotherapy in Children Monosensitized to House Dust Mite?

**DOI:** 10.1155/2012/694094

**Published:** 2012-03-27

**Authors:** Gulbin Bingol Karakoc, Mustafa Yilmaz, Derya Ufuk Altıntaş, Seval Güneşer Kendirli

**Affiliations:** Division of Pediatric Allergy and Immunology, Faculty of Medicine, University of Cukurova, Balcali, 01330 Adana, Turkey

## Abstract

*Background*. Allergen-specific immunotherapy (SIT) is one of the important regimens for the treatment of allergic diseases. Predictive tests for the clinical response to SIT are limited. In this study we aimed to evaluate whether specific IgE/total IgE levels can predict clinical improvement in monosensitized patients to house dust mite treated with immunotherapy. *Patients and Methods*. We analyzed 32 patients who had undergone 2 years of SIT. Serum t-IgE and s-IgE levels, and serum s-IgE/t-IgE ratios were calculated and tested for correlation with clinical response to SIT. Asthma symptom score (ASS), rhinitis symptom score (RSS), pulmonary functions and visual analogue scales (VAS) were evaluated at the beginning and after 2 years. *Results*. There were 17 boys and 15 girls with the mean age of 10.78 ± 3.03 years. The mean serum house dust mite s-IgE level was 128.62 ± 142.61 kU/L, t-IgE 608.90 ± 529.98 IU/mL, and s-IgE/t-IgE ratio 33.83 ± 53.18. Before immunotherapy, ASS was 6.23 ± 1.63, RSS; 8.20 ± 1.88, VAS; 7.38 ± 2.01, FEV1 (%); 89.14 ± 8.48, PEF (%); 88.93 ± 13.57, and after 2 years, these values were determined as 1.90 ± 1.10, 3.05 ± 1.39, 1.35 ± 1.24, 97.6 ± 11.26, and 97.0 ± 11.55, respectively. s-IgE/t-IgE ratio was correlated with change in RSS (*r* = −0.392, *P* = 0.08) and VAS (*r* = −0.367, *P* = 0.05). *Conclusion*. Although SIT is very effective treatment, all patients do not benefit from treatment. We assumed that s-IgE/t-IgE ratio would be useful to predict the clinical response to SIT.

## 1. Introduction

Allergen-specific immunotherapy (SIT) has been used to treat IgE-mediated rhinoconjunctivitis, asthma, and venom hypersensitivity for almost a century. The clinical efficacy of SIT has been shown in several randomized, prospective, double blind placebo-controlled studies [1–5]. A number of studies have shown that SIT decreases clinical symptoms and improves lung function and quality of life in allergic diseases. SIT is only curative and specific method of treatment and may modify the natural course of the allergic disease [3–5]. 

Many complex immunologic changes occur during the course of SIT. Immunological changes induced by SIT are encompassing modulation of allergen-specific antibody responses, reduction in recruitment and activation of proinflammatory cells, and changes in the pattern of allergen-specific T-cell responses [6–9]. It has been postulated that regulatory T (Treg) cells can control an established allergic response via distinct mechanisms. IL-10 and TGF-*β* decrease IgE production and enhance IgG4 and IgA production, respectively [1, 10–12]. Allergen-specific immunotherapy frequently induced a transient initial increase in serum-allergen specific IgE, and a subsequent decrease is observed in the following months or years of SIT [13, 14]. Moreover, successful SIT often elicits allergen-specific IgG4 and IgA responses [6, 15–17]. However, a significant decrease in the allergen-specific IgE/IgG4 ratio occurs after several months. Such changes in the IgE/IgG4 ratio have been found to correlate with a decrease in the late-phase skin reaction to the allergen and with the overall clinical efficacy of SIT in some studies [16–19]. It is unclear how serum total IgE (t-IgE) measurements, alone or in relation to s-IgE measurements, or blood eosinophil (b-eos) counts should be interpreted, as well as what role they should play in selecting patients for SIT in clinical practice [20].

In this study, we aimed to evaluate whether specific IgE/total IgE levels can predict clinical improvement in monosensitized patients to house dust mite treated with immunotherapy. 

## 2. Patients and Methods

We analyzed 32 children monosensitized to house dust mite with the diagnosis of asthma and/or allergic rhinitis that were on followup in Cukurova University, Faculty of Medicine, Division of Pediatric Allergy-Immunology, Adana, Turkey between January 2007 and September 2010. All patients underwent SIT by means of subcutaneous route as part of the therapy.

All patients presented with a clinical diagnosis of asthma and/or allergic rhinitis. Diagnosis of asthma and allergic rhinitis was defined according to GINA and ARIA criteria [21, 22]. Skin prick tests, serum t-IgE and s-IgE levels, and serum s-IgE/t-IgE ratios, asthma symptom scores (ASS), rhinitis symptom scores (RSS), pulmonary functions, and visual analogue scales (VAS) were evaluated at the beginning and after 2 years in all patients.

Immunotherapy was administered by means of subcutaneous route, and each patient received the maximum tolerated dose, as per the manufacturer's recommendations. The maintenance dose was 0.8 mL which corresponds to 3.84 mg of the major allergens of D pteronyssinus and D farinae.

Patients were divided into two groups according to the s-IgE/t-IgE ratio. We set up 16.2 as a cut-off point for s-IgE/t-IgE ratio, and Group 1 included patients who had s-IgE/t-IgE ratio <16.2, while Group 2 s-IgE/t-IgE ratio >16.2 [20].

### 2.1. SPT

A routine skin prick test was performed for all patients using kits containing common inhalant allergens (*D. pteronyssinus*, *D. farinae*, grass mix, tree mix, mold mix, and *Alternaria* species) (ALK-Abello', Madrid, Spain), and an induration with a diameter of ≥3 mm was accepted as a positive reaction.

### 2.2. Total IgE

Total IgE levels were assayed by microparticle enzyme immunoassay (Abott, USA).

### 2.3. spIgE

A blood sample was processed at the time of diagnosis and after 2 years of SIT. s-IgE levels were determined by using the fluoroimmunoassay technique (Unicap, Phadia, Uppsala, Sweden) according to the manufacturer's instructions. Serum s-IgE levels were determined with a detection limit of 0.35 kAU/L. In all patients, the s-IgE level was measured for the same allergens used in the skin prick tests.

spIgE/t-IgE ratio was calculated as

(1) (spIgE/t−IgE)  ×  100.

### 2.4. Asthma Symptom Score (ASS)

Asthma symptom scores were determined based on the daytime symptoms and nocturnal awakening. In addition, the amount of as-needed *β*2-agonist (salbutamol) was recorded daily as the number of puffs. Daytime asthma symptoms and nocturnal awakenings were scored subjectively, as follows: 0, no symptoms during the day/night; 1, symptoms did not affect daily activities or nighttime sleep; 2, symptoms affected at least 1 daily activity or disturbed nighttime sleep; 3, symptoms affected 2 or more daily activities or disturbed sleep all night or most of the night. Use of *β*2-agonists was scored as follows: 0, none; 1, once a day; 2, between 2 and 3 times a day; 3, more than 3 times a day. The minimum score for each day was 0 (no symptoms during the day, no symptoms at night, and no use of *β*2-agonists), and the maximum score was 9 (severe symptoms during the day and at night, and more than 3 administrations of *β*2-agonists) [23].

### 2.5. Rhinitis Symptom Score

Each nasal symptoms including itching, congestion, sneezing, and rhinorrhea were scored subjectively as follows: 0, no symptoms; 1, symptom positive however did not affect daily activities or nighttime sleep; 2, symptoms were bothering but did not affect daily activities or nighttime sleep; 3, symptoms affected daily activities or disturbed sleep. Scores for each symptoms recorded and sum of the scores were calculated (0–12) [24].

### 2.6. VAS (Visual Analogue Scale)

Visual analogue scale was evaluated on 10 cm card [25].

### 2.7. Pulmonary Functions

PFTs were performed according to the American Thoracic Society (ATS) Criteria by using ZAN 100 Spiromed (Oberthulba, Germany), and the results were expressed as percentages of the predicted values for age and height [26].

### 2.8. Determination of Changes in Clinical Parameters

Changes in ASS, RSS, VAS, and pulmonary functions throughout 2 years were calculated by the following formula:

(2) $$\begin{array}{c} \text{Change}\;\text{in}\;\text{ASS}\\ =\;\frac{\text{ASS}\;\left(\text{before}\;\text{treatment}\right)-\text{ASS}\;\left(\text{after}\;\text{treatment}\right)}{\text{ASS}\;\left(\text{before}\;\text{treatment}\right)}\times 100. \end{array}$$

Decreases in RSS, FEV1, PEF, and VAS were calculated with same method.

### 2.9. Statistical Analysis

All statistical analysis was carried out using a computer software (SPSS version 11.0; SPSS; Chicago, Illinois, USA). Nonparametric Wilcoxon test was utilized to compare the groups. Spearman's correlation test was used to determine the statistical relationships between s-IgE/t-IgE ratio and changes in ASS, RSS, and VAS. The *P* < 0.05 value was considered as statistical significant.

## 3. Results

### 3.1. The Characteristics of the Patients

There were 17 boys and 15 girls with the mean age of 10.78 ± 3.03 years (range; 7–15 years). Sixteen patients had asthma, 4 patients had allergic rhinitis, and 12 patients had both asthma and allergic rhinitis. At beginning of the immunotherapy, the mean total IgE value was found to be 608.9 ± 529.987 IU/mL, spIgE and spIgE/t-IgE ratio were 128.62 ± 142.61 kUA/L (median: 69.05) and 33.83 ± 53.18 (median: 17.68), respectively. The characteristics of the patients were given in Table 1. 

### 3.2. Changes in Clinical Symptoms, VAS, and Pulmonary Functions

We found statistically significant improvement in asthma symptom score, rhinitis symptom score, pulmonary functions, and VAS at the end of the 2 years of SIT (Table 2).

### 3.3. Correlation between the s-IgE/t-IgE Ratio and Changes in ASS, RSS, Pulmonary Functions, and VAS

 We analyzed the correlations between the s-IgE/t-IgE ratio and changes in ASS, RSS, pulmonary functions, and VAS in order to determine whether this ratio would be useful to predict the efficacy of SIT. We found that s-IgE/t-IgE ratio was inversely correlated with the change in RSS but this was not statistically significant (*r* = −0.392,  *P* = 0.08). On the other hand, significant inverse correlation was determined between the s-IgE/t-IgE ratio and change VAS (*r* = −0.367,  *P* = 0.05). There was no significant correlation between the s-IgE/t-IgE ratio and changes in ASS and pulmonary functions (Table 3).

In addition, patients were divided into, two groups according to the s-IgE/t-IgE ratio. Previously Di Lorenzo et al. reported that serum s-IgE/t-IgE ratio of greater than 16.2 correlated with an effective clinical response to SIT [20]. Based on this data, we set up 16.2 as a cut-off point for s-IgE/t-IgE ratio [20]. Group 1 included patients who had s-IgE/t-IgE ratio <16.2, while Group 2 included patients with s-IgE/t-IgE ratio >16.2 (Table 4). According to this cut-off point, we found significant inverse correlation between the s-IgE/t-IgE ratio and change in both RSS (*r* = −0.432,  *P* = 0.012) and VAS (*r* = −0.483,  *P* = 0.01). However, there were no significant correlations in Group 1 regarding these parameters. In addition, no statistically significant relation was found between the s-IgE/t-IgE ratio and changes in ASS and pulmonary functions in both groups. 

## 4. Discussion

Allergen-specific immunotherapy has the potential to modify the natural course of allergic diseases [3–5, 27, 28]. Many different in vivo and in vitro tests have been used to determine the efficiency; however, predictive tests for the clinical response to SIT are limited.

Successful immunotherapy is accompanied by the suppression of numbers of T-helper 2 (Th2) effector cells, eosinophils, basophils, mast cells and neutrophils infiltration in target organs, induction of IL-10 and/or TGF-*β* + Treg cells, and increases in “protective” noninflammatory blocking antibodies, particularly IgG4 and IgA2 subclasses with inhibitory activity. This suppression occurs within weeks or months as a consequence of the appearance of a population of regulatory T cells that exert their effects by mechanisms involving cell-cell contact, but also by the release of cytokines such as IL-10 (increases IgG4) and TGF-*β* (increases specific IgA). Mast cell and basophil desensitization are very early effects. Intermediate effects are related to changes in allergen-specific T cells, and late affects are related to B cells and IgE as well as mast cells, basophils, and eosinophils [6]. Several successful immunotherapy studies have shown an induction of peripheral tolerance and an anergic state in activated specific T cells [10–12, 27, 28]. On the other hand, a significant decrease in skin prick test reactivity can be also observed relatively late in the course [3–5]. Therefore, many clinicians want to find out objective and easily performed parameters to predict the efficacy of SIT. Recently, Di Lorenzo et al. demonstrated that the calculation of the serum s-IgE/t-IgE ratio could be useful for predicting the clinical response to SIT offering an advantage over measuring t-IgE and s-IgE levels in monosensitized patients for the following allergens: grass, *Parietaria judaica* [20]. To support this idea, in this study, we evaluate whether s-IgE/t-IgE ratio can predict clinical improvement in monosensitized patients to house dust mite treated with immunotherapy and found that s-IgE/t-IgE ratio would be useful to predict the clinical response to SIT especially in patients with allergic rhinitis. In our study, we demonstrated statistically significant correlation between the s-IgE/t-IgE ratio and change in VAS and RSS. Visual analogue scores and rhinitis symptom scores improved in children with s-IgE/t-IgE ratio higher than 16.2 at the end of the 2 years of SIT.

The continuous hyperreactivity in skin prick test might have negative impact on motivation of both physicians and patients. Therefore, different objective and practical parameters are needed to evaluate the clinical response to SIT. Nasal and/or bronchial provocation tests, diluted skin prick tests, SpIG4 levels, or other detailed tests are usually used in clinical studies or studies aimed to investigate the effectiveness of SIT. However, it is generally difficult to perform all these tests in routine practice because of ethical and economic issues. In this study, we evaluate whether this simple s-IgE/t-IgE ratio can predict clinical improvement in such patients routinely and we assumed that this ratio would be helpful to predict the efficacy of SIT in clinical practice.

Allergen-specific immunotherapy is of long-duration therapy, and at least 3–5-year treatment was recommended for the best clinical benefits [29, 30]. Because not all patients benefit from treatment, it is important to be able to have specific criteria to determine those patients who might best benefit from this therapy and decide when to discontinue SIT. We found that the serum s-IgE/t-IgE ratio would be a good predictor of clinical response to allergen-specific immunotherapy and this ratio might be useful for deciding when to discontinue the SIT as well. However further, studies in larger series are needed.

## Figures and Tables

**Table 1:** The characteristics of the patients.

Characteristics	
Age	10.78 ± 3.03 (7–15 years)
Gender (F/M)	17/15
Diagnosis	*N* (%)
Asthma	16/32 (50%)
Rhinitis	4/32 (12.5%)
Asthma and rhinitis	12/32 (37.5%)
Specific IgE (kuA/L)	128.62 ± 142.61 (median: 69.05)
Total IgE	608.9 ± 529.987
(Specific IgE/total IgE) × 100	33.83 ± 53.18 (median: 17.68)

**Table 2:** The changes in ASS, RSS, pulmonary functions, and VAS following 2 years of SIT.

Parameters	Before SIT	After 2 years of SIT	*P* value
ASS	6.23 ± 1.63	1.90 ± 1.10	0.000
RSS	8.20 ± 1.88	3.05 ± 1.39	0.000
FEV1	89.14 ± 8.48	97.60 ± 11.26	0.000
PEF	88.93 ± 13.57	97.0 ± 11.55	0.001
VAS	7.38 ± 2.01	1.35 ± 1.24	0.000

**Table 3:** Correlations between s-IgE/t-IgE ratio and change in ASS, RSS, pulmonary functions, and VAS.

Correlations	*R* value	*P* value
ASS	−0.115	0.54
RSS	−0.392	0.08
FEV1	0.215	0.281
PEF	0.136	0.498
VAS	−0.367	0.05

**Table 4:** Correlations between s-IgE/t-IgE ratio and change in ASS, RSS, pulmonary functions and VAS based on cut off value 16.2 for s-IgE/t-IgE ratio.

	Correlations	*R* value	*P* value
GROUP 1 (s-IgE/t-IgE ≤16.2) *N* = 16	ASS	0.239	0.410
	RSS	0.116	0.780
	FEV1	0.121	0.694
	VAS	0.124	0.296

GROUP 2 (s-IgE/t-IgE >16.2) *N* = 16	ASS	−0.173	0.078
	RSS	−0.432	**0.012**
	FEV1	0.249	0.091
	VAS	−0.483	**0.01**
